# Secure Triggering Frame-Based Dynamic Power Saving Mechanism against Battery Draining Attack in Wi-Fi-Enabled Sensor Networks

**DOI:** 10.3390/s24165131

**Published:** 2024-08-08

**Authors:** So-Yeon Kim, So-Hyun Park, Jung-Hoon Lee, Il-Gu Lee

**Affiliations:** 1 Department of Future Convergence Technology Engineering, Sungshin Women’s University, Seoul 02844, Republic of Korea; 220237014@sungshin.ac.kr (S.-Y.K.); 220227022@sungshin.ac.kr (S.-H.P.); 2 Department of Electronics Engineering and Applied Communications Research Center, Hankuk University of Foreign Studies, Yongin 17035, Republic of Korea; tantheta@hufs.ac.kr

**Keywords:** multi-link operation (MLO), battery draining attack, secure triggering frame, dynamic power saving mechanism, energy efficiency, latency, security

## Abstract

Wireless local area networks (WLANs) have recently evolved into technologies featuring extremely high throughput and ultra-high reliability. As WLANs are predominantly utilized in Internet of Things (IoT) and Wi-Fi-enabled sensor applications powered by coin cell batteries, these high-efficiency, high-performance technologies often cause significant battery depletion. The introduction of the trigger frame-based uplink transmission method, designed to enhance network throughput, lacks adequate security measures, enabling attackers to manipulate trigger frames. Devices receiving such frames must respond immediately; however, if a device receives a fake trigger frame, it fails to enter sleep mode, continuously sending response signals and thereby increasing power consumption. This problem is specifically acute in next-generation devices that support multi-link operation (MLO), capable of simultaneous transmission and reception across multiple links, rendering them more susceptible to battery draining attacks than conventional single-link devices. To address this, this paper introduces a Secure Triggering Frame-Based Dynamic Power Saving Mechanism (STF-DPSM) specifically designed for multi-link environments. Experimental results indicate that even in a multi-link environment with only two links, the STF-DPSM improves energy efficiency by an average of approximately 55.69% over conventional methods and reduces delay times by an average of approximately 44.7% compared with methods that consistently utilize encryption/decryption and integrity checks.

## 1. Introduction

Recently, the wireless local area network (WLAN) has expanded from everyday applications to various industrial fields, necessitating the handling of large traffic volumes and real-time ultra-low latency [1,2]. The throughput requirements for streaming 4K or 8K video approach 20 Gbps, with a notable increase in ultra-high throughput and stringent low-latency applications such as virtual reality (VR) and augmented reality (AR) [3]. As WLANs are predominantly used in battery-powered, resource-constrained devices, advanced WLAN technologies can lead to significant battery consumption [4]. In Wi-Fi-enabled sensor networks that connect various sensors using Wi-Fi technology for data collection and processing, it is important to increase battery life because IoT devices and sensors powered by coin cell batteries need to communicate and interact [5,6]. Consequently, WLAN standards have incorporated power-saving technologies such as power saving mechanisms (PSMs), target wake time (TWT), and wake-up radio (WuR) to optimize performance and battery efficiency [7,8,9,10,11,12].

The 802.11ax standard [13] focuses on efficient resource utilization, evolving from the 802.11ac, which only supported downlink (DL) multi-user (MU) multiple-input multiple-output (MIMO) [14,15]. It now includes support for uplink (UL) MU-MIMO to enhance network throughput. A trigger-based operation has been introduced to schedule UL MU-MIMO, with the High-Efficiency Trigger-Based PLCP Protocol Data Unit (HE TB PPDU) being defined for this purpose. Furthermore, the 802.11be standard [16] introduces multi-link operation (MLO), allowing a single device to transmit and receive data simultaneously using multiple links, achieving ultra-high throughput performance [17].

While trigger frames and multi-link operation ensure high performance and efficient network throughput, they also introduce security threats that expose the battery vulnerabilities of low-power devices. The absence of protective measures for devices receiving trigger frames and providing uplink feedback makes them susceptible to malicious trigger frame attacks. Next-generation devices operating with multiple links are more prone to battery draining attacks than conventional single-link devices. This study aims to analyze the methods and vulnerabilities of attacks exploiting trigger frames in multi-link devices (MLD) and proposes a Secure Triggering Frame-Based Dynamic Power Saving Mechanism (STF-DPSM) to counter these threats.

The main contributions of this paper are as follows:

- This paper analyzes the structural vulnerabilities of trigger frames in trigger-based uplink transmission methods and proposes methods to combat battery draining attacks in a multi-link environment.
- We propose the STF-DPSM, which combines dynamic power-saving methods to enhance energy efficiency with a secure trigger frame (STF) to counter power-draining attacks while ensuring confidentiality and integrity.
- We propose a performance evaluation framework for power-saving methods designed to counter trigger-based battery draining attacks in next-generation Wi-Fi-enabled sensor networks.

The structure of this paper is as follows: Section 2 analyzes the security vulnerabilities of WLANs and identifies previous studies related to battery draining attacks. Section 3 details the multi-link operating environment, the method of trigger frame-based battery draining attacks, and the operational principles of this study. Section 4 describes the defense mechanism to respond to battery draining attacks, and Section 5 presents the results of a trigger frame-based attack simulation on a multi-link device. The study concludes in Section 6.

## 2. Security Vulnerability in 802.11

### 2.1. Main Features up to 802.11ac

Wireless LAN has developed significantly, ensuring compatibility with previous standards while enhancing communication throughput and efficiency [18]. In the quest for high throughput (HT), the 802.11n standard [19] marked the first introduction of multiple input multiple output (MIMO) technology to wireless LANs, enabling high-speed transmissions of up to 600 MHz in both the 2.4 GHz and 5 GHz frequency bands [20,21]. MIMO utilizes multiple antennas at both the transmitting and receiving ends to form multiple spatial streams, thereby achieving spatial diversity effects and enhancing the receiver’s signal-to-noise ratio (SNR) [22]. In the 802.11n standard, both transmitting and receiving ends could be equipped with up to four antennas, allowing the use of up to four spatial streams. Additionally, this standard supported transmit beamforming to improve downlink throughput performance [23].

The 802.11ac standard [24] introduced Gigabit Wi-Fi technology, targeting speeds up to 6 Gbps in the 5 GHz frequency band [25,26]. It expanded the frequency bandwidth from 20 and 40 MHz to 80 and 160 MHz, significantly boosting throughput. Notably, the introduction of downlink (DL) multi-user MIMO (MU-MIMO) enabled the multiplexing of multiple antennas on a single access point (AP) for individual users, efficiently enhancing the overall system throughput performance [27].

### 2.2. Trigger Frame in IEEE 802.11ax

The 802.11ax standard aims to maximize resource efficiency and communication speed, primarily targeting high-efficiency wireless networking in dense environments by supporting multi-user techniques for both downlink and uplink transmissions [28]. A central technology of 802.11ax is orthogonal frequency division multiple access (OFDMA), which segments the subcarriers of a channel into resource units (RUs) and assigns different RUs to each station (STA), enabling simultaneous usage of the same frequency resource. DL OFDMA enables one AP to transmit data to multiple STAs concurrently, while UL OFDMA enables an AP to receive data from multiple STAs simultaneously on the same frequency channel [29].

The 802.11ax standard can support up to eight users on a single RU via MU-MIMO [30]. Unlike the prior 802.11ac standard, which supported only DL MU-MIMO, 802.11ax introduces UL MU-MIMO technology, thereby boosting network throughput performance [14]. The implementation of UL MU-MIMO technology incorporates a trigger frame-based multi-user channel access mechanism to schedule UL transmissions from multiple user stations to a single AP. To avoid collisions among multiple users, MU-RTS (Ready to Send)/CTS (Clear to Send) technology is utilized [31].

In the context of 802.11ax, the trigger frame is initiated by the AP and directed to multiple STAs to coordinate uplink channel access. The trigger frame’s fields include a list of participating STAs, relevant RU allocation information for each STA, and details about transmission duration [32]. Upon receiving the AP’s trigger frame, the STA responds by transmitting data to the AP.

### 2.3. Multi-Link Operation (MLO) in IEEE 802.11be

The recent 802.11be amendment, which aims to achieve Extremely High Throughput (EHT), introduces multi-link operation (MLO) as a key feature [33]. This technology allows a single AP and station (STA) to operate across multiple bands or channels simultaneously. In a multi-link device, whether it is functioning as an AP or STA using MLO, the media access control (MAC) sub-layer is crucial. Within a single AP/STA MLD, the structure includes several components: affiliated AP/STAs, logical link control (LLC), upper MAC (U-MAC), and low MAC (L-MAC). These components are responsible for managing and processing data across the various links of affiliated AP/STAs [34,35].

### 2.4. Vulnerabilities of Trigger Frame in MLO

The use of trigger-based UL MU-MIMO significantly enhances the network throughput of wireless LANs. However, vulnerabilities arise if the field information within trigger frames is tampered with, exposing the network to potential security threats such as replay attacks and flooding denial-of-service (DoS) attacks [36]. For rapid data transmission control, the fields of the control frame header in WLANs were transmitted unencrypted. This vulnerability allowed for the possibility of sending abnormal trigger frames with manipulated address information, which could intentionally disrupt service availability of STAs by eliciting continuous responses and consequently draining their batteries [37,38]. Furthermore, with the advent of multi-link operation introduced in the 802.11be amendment, the implications of attacks utilizing trigger frame-based UL MU-MIMO for uplink multiuser transmission could be more severe compared to devices using conventional single links. Therefore, securing trigger frame-based transmission operations becomes crucial to mitigate these potential security risks and maintain the integrity and efficiency of the network.

### 2.5. Related Work

Wireless LAN MAC frames are typically transmitted without encrypting the header portion to ensure fast and accurate data transmission control. Consequently, malicious actors can intentionally induce UL transmissions from receiving STAs by manipulating or replaying MAC frame headers, ultimately disrupting STA availability and leading to battery depletion. This section outlines potential battery draining attacks that can occur in conventional Wi-Fi-enabled networks, as shown in Table 1.

Due to the heightened susceptibility of low-power devices, such as those in the Internet of Things (IoT), to battery depletion attacks, extensive research has been conducted to analyze the types and impacts of these attacks in low-power wireless (LPW) networks and wireless sensor networks (WSNs) [39,40,41,42]. Particularly, low-power devices employ mechanisms such as TWT and wake-up radio (WUR) within beacon frames to conserve battery power, awakening from a low-power state upon receiving trigger messages. However, vulnerabilities in these operations can be leveraged for battery draining attacks [43,44,45].

**Table 1:** Previous studies on battery drain attacks in Wi-Fi-enabled networks.

Ref.	Attack Vector and Scenario	Countermeasure		Limitation
		Detection	Response	
[40]	Conducting five different battery drain attacks, including packet flooding	-	-	No mechanisms exist to detect and respond to battery drain attacks
[41]	Repeatedly sending fake messages with manipulated MAC frame counter values to consume significant energy in verification by the receiver	Detecting battery depletion attacks through an energy prediction-based intrusion anomaly detection system	Introduction of frame encryption	The encryption method induces high overheads during normal operation, degrading network performance
[42]	Attacker poses as a legitimate node to perform energy drain attacks	By detecting abnormal packet transmission intervals, the attack node is temporarily blocked	Verify the integrity and authentication of packets using symmetric and asymmetric encryption	Increased system complexity due to the introduction of encryption and security mechanisms
[43]	Battery drain attack through unencrypted TWT negotiation procedures	By measuring the power consumed in a normal state, a power consumption model is established. This model is used to detect abnormal power consumption caused by attacks	By encrypting the TWT negotiation procedure and improving the scheduling algorithm, power consumption and security issues are addressed	Encrypting the TWT negotiation procedure increases system complexity and leads to performance degradation
[44]	Sending unauthorized packets or replaying recorded traffic to prevent the device from entering sleep mode, causing a sleep deprivation attack	During the training period, the system learns the normal state of the network. If an abnormally high number of wake-up packets are received during the transmission opportunity, it is considered an attack	Notify the AP of the attack and allocate a new wake-up ID with the AP to prevent the attack	There are issues with increased complexity and false positive rates. Additional authentication processes may cause overheads
[45]	Performing a battery drain attack by forging beacon signals with manipulated timestamps and TIM elements to make the client send unnecessary frames	Add authentication tags to beacon frames to detect forged beacons and report the alert to the AP	Extend the 802.11 standard [46] to authenticate beacon frames	The process of reporting to the AP and adding authentication tags introduces overhead issues
[47]	Performing a sleep deprivation attack by continuously transmitting specially crafted wireless signals near the sensor to prevent IoT devices from entering sleep mode	Collect real-time battery consumption data from sensors and detect based on threshold values	Sensors automatically switch to power-saving mode if battery consumption exceeds the threshold	Switching to power-saving mode causes availability issues, as sensors cannot communicate for a certain period
[48]	Attacker performs a battery drain attack by sending a large number of packets at shorter intervals than the actual sensor node	Detect an attack if packets are transmitted more frequently than the configured transmission interval	Monitor the transmission intervals of nodes to detect abnormal activities and temporarily block the attack node	There is the possibility of false positives, and the process of detecting and blocking attacks consumes additional energy and resources
[49]	Manipulating specific information elements in beacons to cause battery drain or changing TIM elements to prevent IoT devices from entering sleep mode	Use packet analysis tools and battery monitoring tools to detect attacks	Verify the integrity of beacon frames using a beacon integrity group temporal key (BIGTK)	The introduction of integrity verification mechanisms increases system complexity
[50]	Attacker performs a DoS attack by spoofing IP addresses and sending a large number of messages, draining the IoT device’s battery	Detection by verifying the validity of messages based on short message authentication codes	Defending against attacks through cooperation with the gateway. The gateway filters attack messages, and the IoT device handles only legitimate requests	Dependent on the performance and reliability of the gateway
[51]	Attacker drains the energy of intermediate nodes by sending a large number of packets	Each node records the number of packets received within a specific time frame and compares it to a dynamically calculated threshold to detect potential attacks	Isolate suspected malicious nodes from the network to prevent them from receiving or transmitting any packets	Additional resource consumption occurs during the threshold calculation and comparison process, and attackers may find ways to bypass it
[52]	Attacker inserts malformed packets to force the target device to consume unnecessary energy	Detect attacks by analyzing the CRC error patterns of received packets	Set transmission and reception times randomly to prevent attackers from predicting packet insertion times. Additionally, if a faulty packet is detected, stop receiving packets on the corresponding channel or time slot to avoid additional energy consumption	The threshold set for attack detection increases implementation complexity
[53]	Attacker depletes the device’s battery through continuous service requests	Analyze the MAC header to identify error patterns	When abnormal requests are detected, limit those requests to minimize energy consumption	Detection becomes difficult if attackers evade error patterns
[54]	Attacker continuously sends unauthorized fake packets, causing the device to constantly respond and deplete its battery	Monitor abnormal Ack response patterns caused by fake packets	Add integrity checks to beacon frames to detect and ignore fake beacon frames	Integrity checks lead to complexity issues and performance degradation
Our Work	Exploiting the vulnerability of trigger frames to make multi-link devices continuously consume energy	The normal AP detects masquerading attacks	Adjust the power-saving time adaptively, and use the STF method, which applies security only after an attack has occurred using trigger frames	Lack of evaluation in realistic environments

Smith et al. [40] analyzed the battery vulnerabilities of IoT devices, which often prioritize lightweight security measures due to their nature. They demonstrated packet flooding attacks exploiting the characteristic of IoT nodes responding to destination-oriented directed acyclic graph (DODAG) information solicitation (DIS) messages after receiving DODAG information object (DIO) messages, ultimately leading to the rapid depletion of IoT device batteries. They also conducted attacks, such as blackhole attacks and rank attacks, indicating the possibility of battery draining attacks using specific insecure messages in low-power IoT networks.

Nguyen et al. [41] systematically demonstrated the potential for energy draining attacks (EDAs) in low-power wireless (LPW) networks, such as Zigbee, 802.11ah, Bluetooth Low Energy (BLE), NB-IoT, and LTE-M, facilitated by unencrypted PHY/MAC layers and insecure network protocols. They explained that attacks such as jamming and communication in hidden scenarios are possible in the PHY layer, while attacks such as garbage data verification and duty-cycle manipulation (leading to sleep or depletion) can occur in the MAC layer.

Tropea et al. [42] proposed cryptographic techniques to address battery depletion and impersonation attacks in wireless sensor networks (WSNs). They evaluated the performance of each encryption method by applying Advanced Encryption Standard (AES), Rivest-Shamir-Adleman (RSA), and Elliptic Curve Cryptography (ECC) to lightweight MAC (LMAC) and Berkeley MAC (BMAC), which are MAC protocols used in WSNs, to mitigate various attacks.

Liu et al. [43] evaluated the impact of the new channel access mechanisms in 802.11ax, namely, OFDMA and TWT, from the perspectives of throughput and energy consumption. They found that, compared with the legacy CSMA/CA scheme, Wi-Fi 6 networks offer a threefold increase in throughput and a fivefold decrease in latency. However, while OFDMA brings benefits in terms of throughput and latency, it also introduces increased energy consumption costs. They highlighted that the TWT negotiating procedure is vulnerable to malicious attacks, such as battery depletion, packet loss, and throughput-throttle attacks. They compared normal traffic with traffic under TWT attack scenarios to evaluate the impact of attacks from a throughput perspective.

Park et al. [44] proposed the anti-malicious attack (AMA-WUR) algorithm to address non-sleep attacks in the power-saving protocol of 802.11ba wake-up radio (WUR) designed to minimize the power consumption of IoT devices. The WUR protocol involves the AP sending a wake-up packet (WUP) when data transmission is needed, causing the receiving STA to wake up and enter a state ready to receive data. To detect DoS WUP-based attacks, the WUP utilization rate during TXOP intervals was used to identify attacks. Additionally, they proposed a method to utilize the reserved bit in the MAC header to transmit detected flooding attack results and respond to attacks accordingly.

Vanhoef et al. [45] proposed the broadcast integrity protocol (BIP) as a pre-authentication method for beacon frames to address the possibility of attacks where malicious actors forge Wi-Fi beacon frames. The BIP involves the AP generating a symmetric key and authenticating beacons, preventing external devices from forging beacon frames.

The trigger frame is a frame that causes the AP to initiate data transmission to the STA. This frame is likely to be exploited in battery consumption attacks; however, insufficient research has been conducted on the security of the trigger frame. Additionally, with the introduction of 802.11be, multi-link-based operation becomes possible, which could further amplify the ripple effect of trigger-based battery depletion attacks. Despite this, few studies have analyzed the impact of the trigger frame on secure trigger frame behavior and the possibility of attacks in multi-link devices. Therefore, this study analyzes the influence of the trigger frame from the perspective of energy consumption and proposes a protection technology for secure UL MU transmission in multi-link devices, which aligns with the direction of WLAN power management.

## 3. Triggering-Based Battery Draining Attacks

**Figure 1:**
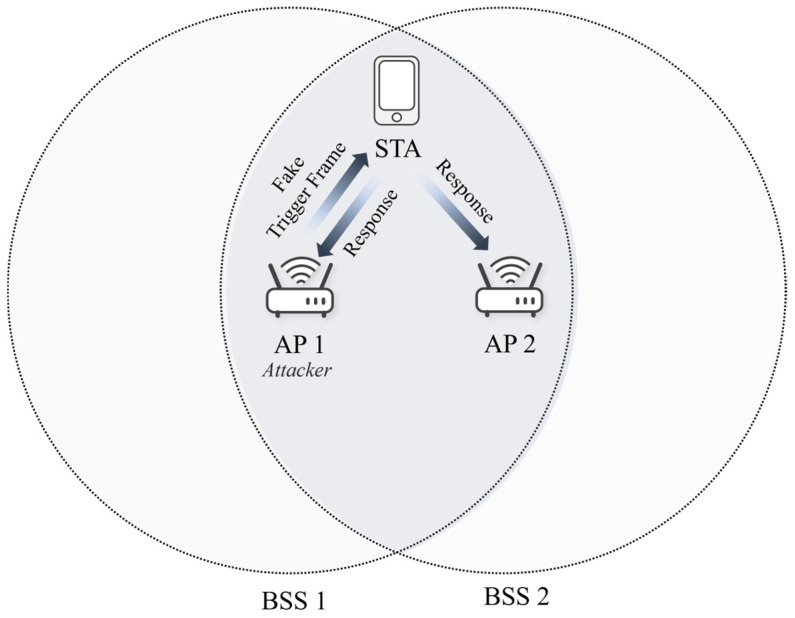
Network configuration for battery draining attack.

This study explores the vulnerability of trigger frames in a multi-link environment to conduct battery draining attacks. Figure 1 depicts the network configuration used for battery draining attacks where the coverage of APs overlaps. In this overlapped basic service set (OBSS) environment, STA MLDs and AP MLDs operate with the capability to configure multi-links, each link potentially utilizing the 2.4 GHz, 5 GHz, or 6 GHz bands. Multiple links are feasible within a single band. Moreover, each link of the STA operates in simultaneous transmit and receive (STR) mode, where each link independently functions without interfering each other.

The trigger-based battery depletion attack incorporates a masquerading attack, where the attacking AP, denoted as AP 1, masquerades as a legitimate AP to deceive wireless terminals, combined with a DoS attack technique to drain the resources of wireless terminals. Initially, AP 1 deceives the STA by spoofing the MAC address or service set identifier (SSID) of the legitimate AP (AP 2), causing the STA to recognize AP 1 as AP 2. While masquerading as AP 2, AP 1 sends fake trigger frames to the STA. Believing these frames are from AP 2, the STA responds by sending response signals directed to AP 2. AP 1 exploits the vulnerability in trigger frames, which lack security measures and compel the receiving device to respond immediately upon reception. Consequently, if AP 1 continuously dispatches forged trigger frames, the STA is unable to enter power-saving mode and persistently transmits response signals, thereby increasing power consumption.

Legacy wireless terminals typically operate in single-link mode, managing simultaneous transmission and reception on a single channel. In such scenarios, the victim terminal is unable to process regular service traffic during battery draining attacks, as it is occupied responding to triggered signals. Moreover, with the advent of multi-link operation in the 802.11be standard, which supports at least two or more links, the severity of battery draining attacks escalates with the increase in the number of links. Consequently, countermeasures are needed to prevent trigger-based battery draining attacks by ensuring the integrity of trigger frames.

## 4. Proposed STF-DPSM

This section introduces the STF-DPSM as a countermeasure against battery draining attacks. The key components of the STF-DPSM include the DPSM technique, which adaptively adjusts the power saving times based on the frequency of attacks, and the STF structure, which ensures secure triggering transmissions through the use of encryption/decryption methods and integrity check techniques. Most previous research has primarily proposed the introduction of encryption/decryption schemes or integrity assurance methods to protect frame headers as a response to battery draining attacks. However, these methods have the limitation of causing overheads in normal situations without attacks, thereby degrading network performance. Therefore, this paper proposes a method that first adjusts the power-saving time and then uses the STF to address the trade-off between security and efficiency.

**Figure 2:**
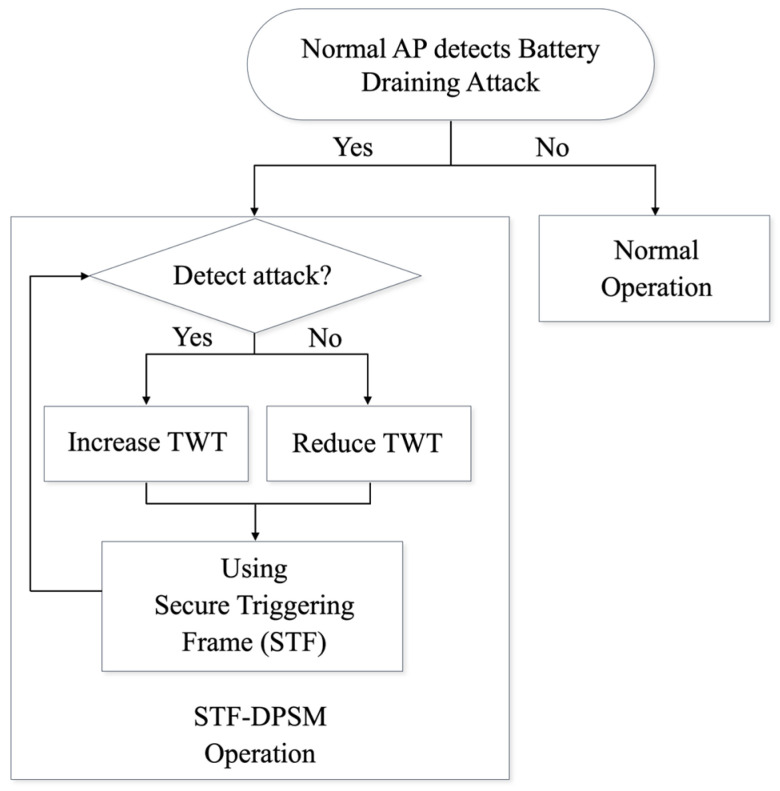
Flowchart of STF-DPSM.

The operational process of the STF-DPSM is depicted in Figure 2. When a legitimate AP detects a battery draining attack, it activates the STF-DPSM mechanism. This involves the AP negotiating the TWT of the victim device through available links. If further battery draining attacks are detected, the TWT period for the victim device is extended during the negotiation phase. Conversely, if no subsequent attacks occur, the TWT period is decreased. Throughout this period, the STF is employed to verify the integrity of the trigger frames. The STF-DPSM dynamically sets the sleep mode duration for the device and defends against battery draining attacks by using secure frames specifically in environments prone to such attacks. This strategy not only enhances energy efficiency but also minimizes operational overheads, making it an effective tool against the threats posed in wireless networks.

**Figure 3:**
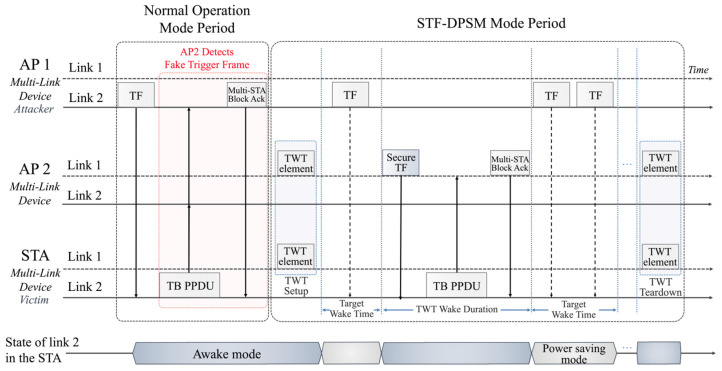
Timing diagram of STF-DPSM.

The detailed operational process of the STF-DPSM is illustrated in Figure 3 for enhanced simplicity and clarity. In this figure, AP 1, AP 2, and STA are shown as multi-link devices, each supporting two links. During the Normal Operation Mode, uplink transmissions are executed using conventional trigger frames. The process of conventional trigger frame-based uplink transmission involves the following steps: firstly, the AP sends trigger frames to all STAs. Upon receiving these frames, STAs verify the information to determine their participation. Secondly, STAs synchronize their frequency, transmission time, and power levels to prevent interference with other STAs. Once synchronization is achieved, STAs transmit the TB PPDU, and the AP acknowledges successful reception with a Block Ack.

As discussed in Section 3, conventional trigger frames lack robust security mechanisms, leading to situations where STAs continuously send response signals upon receiving trigger frames. This vulnerability can result in battery draining attacks involving fake trigger frames during normal operation mode.

When a legitimate AP identifies such fake trigger frames, it switches to STF-DPSM mode. STF-DPSM mode comprises several phases:

- TWT Setup or Teardown Phase: This initial phase involves adjusting the TWT settings based on the detection of abnormal trigger activities to either prolong or terminate the TWT.
- Target Wake Time: During this phase, the device remains in a power-saving state to minimize power consumption. This interval is crucial for conserving battery life, particularly in environments susceptible to attack.
- TWT Wake Duration: In this segment, the device exits the power-saving state to perform data transmission and reception at the scheduled TWT.

This segmented approach in STF-DPSM mode ensures that devices only operate in high-power modes during necessary periods, significantly reducing the risk of battery depletion while maintaining the integrity and security of wireless communications.

TWT is a power-saving mechanism introduced in the 802.11ax standard, designed to reduce energy consumption by negotiating the frequency of data transmissions, allowing devices to stay in a power-saving state for extended periods rather than remaining constantly active. Stations (STAs) activate only during designated data transmission and reception periods, conserving energy and reducing the presence of competing devices within the same basic service set (BSS), which decreases collisions and contention. TWT operates in two modes: broadcast and individual. In broadcast mode, the AP schedules the TWT for participating STAs by broadcasting the TWT element. In individual mode, STAs exchange TWT elements during the setup and agreement process to set their TWTs individually. The TWT element includes management frames such as beacons and probe responses.

**Figure 4:**
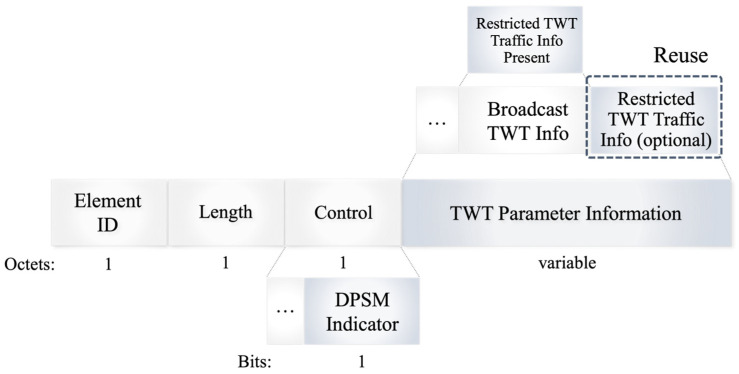
TWT element format.

This paper utilizes broadcast TWT to implement a dynamic power-saving mechanism (DPSM). The structure of the TWT element, as depicted in Figure 4, comprises several fields: an Element ID field (1 octet) that identifies the type of TWT element, a Length field that indicates the length of the TWT element, a Control field (1 octet), and a variable-length TWT Parameter Information field. DPSM employs the reserved lower 1 bit of the Control field to define the DPSM indicator. When set to 1, the Restricted TWT Traffic Info Present field in the lower Broadcast TWT Info field of the TWT Parameter Information is set to 0. This field is typically used by routers to reserve bandwidth for specific data types but is repurposed here to assign the link ID to a power-saving state. This reuse is vital, especially since setting up TWT on a link under attack could result in interference due to continuous triggering signals characteristic of battery draining attacks.

During the TWT setup or teardown phases, the legitimate AP multi-link device (AP MLD) engages in TWT negotiations with the victim STA MLD using available links, thereby activating STF-DPSM mode. In this process, the STA MLD negotiates the TWT for the attacked link and dynamically adjusts the TWT duration based on the current threat level. If the negotiation is successful, the attacked link transitions to a power-saving state with parameters set during the negotiation. Throughout the TWT wake duration period, the integrity of the trigger frames is verified using the STF, which incorporates integrity check techniques and encryption technologies. This validation process prevents the STA MLD from indiscriminately responding to trigger frames, thereby conserving energy and disrupting the continuity of the battery draining attack. Finally, once the negotiated TWT period concludes, STF-DPSM mode is terminated by exchanging TWT elements again, returning the system to its regular operational state.

**Figure 5:**
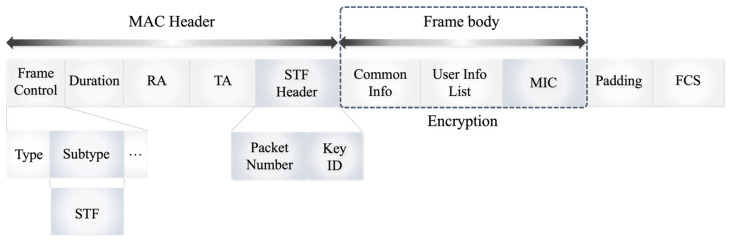
Secure trigger frame format.

The frame structure of the STF is depicted in Figure 5. In conventional wireless communication, control frames facilitate the smooth transmission and reception of data frames, which are typically segmented into the MAC header, frame body, and frame check sequence (FCS). The MAC header contains critical elements such as the frame control field, which includes frame type, transmission speed, and other control information, the Duration field, indicating the expected time for frame transmission, and the receiver address (RA) and transmitter address (TA) fields. Additionally, the frame control field contains a 4-bit Subtype field that specifies the exact frame type.

The frame body houses the necessary information for the frame’s intended function, while the FCS performs an integrity check to confirm the frame has not been tampered with during transmission. Padding fields may also be used to adjust the frame size as required. Conventional trigger frames, which allocate resources for uplink transmissions from multiple users, further include Common Info and User Info List fields. The Common Info field encompasses essential network-wide information, such as the expected length and transmission bandwidth of response frames for uplinks. The User Info List field provides detailed specifications for each STA involved in the uplink transmission, such as RU allocation and coding type.

The proposed STF framework enhances security by using one of the reserved subtype fields (0110, 0111, or 1111) in the frame control field specifically for STF applications. This includes an additional STF header within the MAC header and a message integrity code (MIC) field, a hash-based authentication code for integrity checking, positioned before the FCS and Padding fields. The frame body’s Common Info field, User Info List field, and MIC are encrypted to safeguard the confidentiality and integrity of the trigger frame.

The STF header features a packet number, serving as a unique identifier for each packet, and a key ID subfield, which is employed to identify the encryption key. By combining the packet number and key ID, an encryption key specific to the packet is generated. This key, along with the data from the Common Info and User Info fields, is used to create a hash value to compute the MIC. When the STF is transmitted, the receiving terminal generates a decryption key from the subfields in the STF header, decrypts the message, and performs an integrity check. The receiving terminal recalculates the MIC using the same hash function and key and compares it with the received MIC to verify the integrity of the trigger frame. This method ensures that if a terminal cannot generate the decryption key from the STF header, it will not be able to decode the encrypted trigger frame, thereby maintaining the confidentiality and integrity of the data and protecting it from attackers.

## 5. Evaluation Results and Analysis

This section assesses energy consumption per link based on attack intervals and compares the energy efficiency and latency between conventional methods and the STF-DPSM under various proportions of attack traffic within the total generated traffic. Both models—conventional and proposed—are assumed to operate in environments with two and four links. The conventional method, which lacks security mechanisms, remains continuously active, regardless of the nature of the traffic (regular or attacked). In contrast, the proposed STF-DPSM is tested in two scenarios: one where the STF (with integrity check and encryption) is consistently employed whenever traffic occurs, and another where the DPSM dynamically adjusts the power-saving time for the affected device upon attack detection, using the STF only during the TWT wake duration. In this case, the battery draining attack occurs in the overlapping area of the OBSS environment, and the normal AP detects the attack.

The TWT setting increases exponentially as a power of 2, with the maximum TWT value being determined by the parameter α, for which values of 10 and 100 are considered in this study. The system parameters, including these settings, are summarized in Table 2. The system parameters reflect the parameter values used in commercial Wi-Fi devices to mirror actual network environment elements. The traffic is fixed at 1000 bytes, and the experiments are conducted 1000 times to measure the average time and energy consumption associated with transmitting and receiving the total generated traffic. Energy consumption is calculated based on the electrical energy (Joules) used over time, taking into account the voltage and current. The energy efficiency is then evaluated using Equation (1), which calculates the energy consumed per unit time.

In these experiments, the voltage V is fixed at 1.1 V. Total energy consumption is calculated by multiplying the time spent in each device state (TX for transmitting, RX for receiving, IDLE for standby, and DOZE for power-saving mode) by the power consumed in each respective state. Energy efficiency is subsequently computed by dividing the total energy consumption by the time taken to transmit and receive the total generated traffic. Notably, the TX state is the most power-intensive, while the DOZE state consumes the least amount of power.

Average latency, another critical metric, is evaluated by measuring the time it takes for a packet to be received and processed.

(1) $$Energy\;Efficiency\;\left(J∕s\right)=\frac{P_{TX}T_{TX}+P_{RX}T_{RX}+P_{IDLE}T_{IDLE\;}+P_{DOZE}T_{DOZE\;}}{T_{TOTAL}}$$

**Figure 6:**
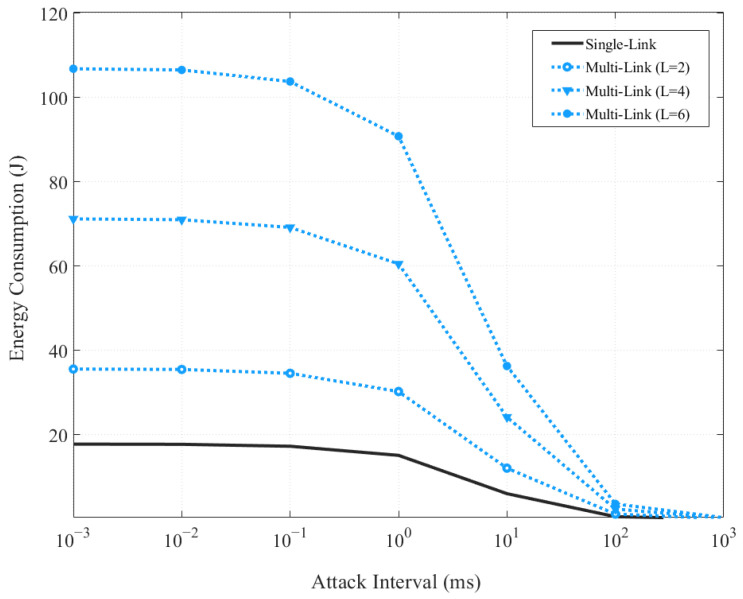
Energy consumption per link based on attack intervals.

Figure 6 presents the energy consumption per link as a function of attack intervals, with the parameter L representing the number of links. In scenarios lacking a defense mechanism, the data illustrate that energy consumption escalates notably with an increase in the number of links in a multi-link setup compared to a single-link configuration. Additionally, this consumption is further exacerbated as the attack intervals narrow, leading to increased energy demands. The rise in energy consumption can be attributed to the additional energy required to activate more links and manage the data transmission or reception across these links. More links in operation mean more pathways that need energy for activation and maintenance. Furthermore, narrower attack intervals result in more frequent activations due to attack signals, compounding the energy requirements. For instance, when the attack interval is 10^−3^ and L is 2, the energy consumption in a multi-link scenario is approximately 50% higher than in a single-link setup. With L increasing to 4, the energy consumption rises by approximately 70%. More dramatically, with L at 6, the increase surges to approximately 500%. These figures starkly illustrate the significant impact that both the number of operational links and the frequency of attack intervals have on energy consumption in the absence of any defensive mechanisms.

**Figure 7:**
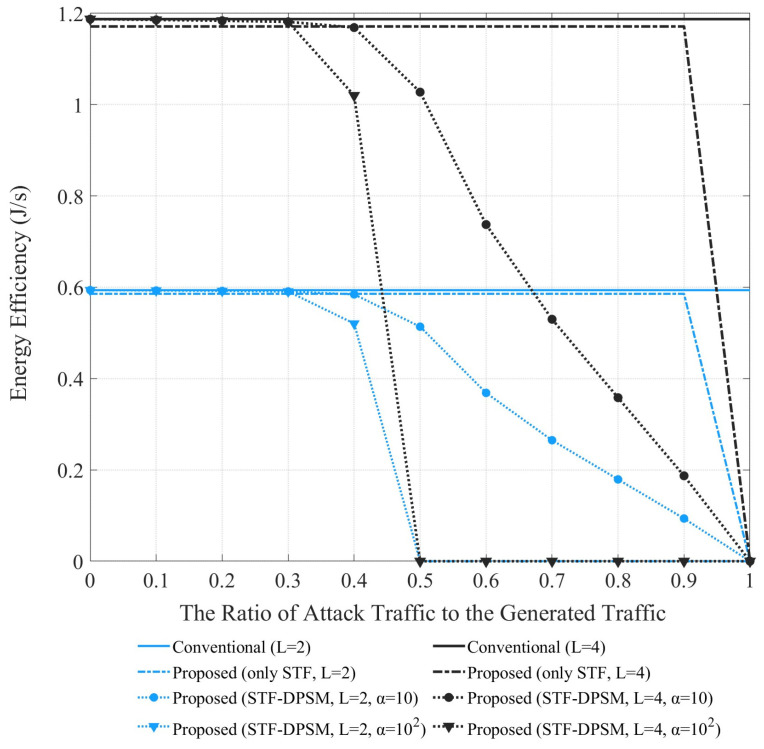
Energy efficiency based on the proportion of attack traffic in the total generated traffic.

Figure 7 shows the energy efficiency as influenced by the proportion of attack traffic within the total generated traffic. The conventional method, which lacks any form of defense logic, exhibits constant energy efficiency across varying link numbers (parameter L). This constant performance is attributed to its mechanism of triggering traffic signals whenever traffic occurs, which generally results in low energy efficiency due to the lack of selective activation or deactivation of links based on traffic nature. In contrast, the proposed method employing only the STF exhibits a slight improvement in energy efficiency, averaging about 1.36% in an environment with L = 2. However, the STF-DPSM method, which integrates the DPSM and STF, shows a marked improvement in energy efficiency as the proportion of attack traffic increases. The parameter α, representing the maximum value of TWT, highlights significant variations based on its settings: in environments with a larger α (=100), energy efficiency improves dramatically as attack traffic increases, due to the extended periods in the power-saving state allowed by a larger α value, effectively reducing energy consumption. Conversely, with a smaller α = 10, energy efficiency improvement is more gradual. In an environment with L = 2 and α = 10, the STF-DPSM method enhances energy efficiency from a minimum of 0.15% to a maximum of 84.25% compared with the conventional method and demonstrates an average improvement of approximately 25% over the method that consistently uses the STF. For L = 2 and α = 100, the improvements in energy efficiency average about 55.69% and 50.59% compared to the conventional method and the consistently used the STF method, respectively.

**Figure 8:**
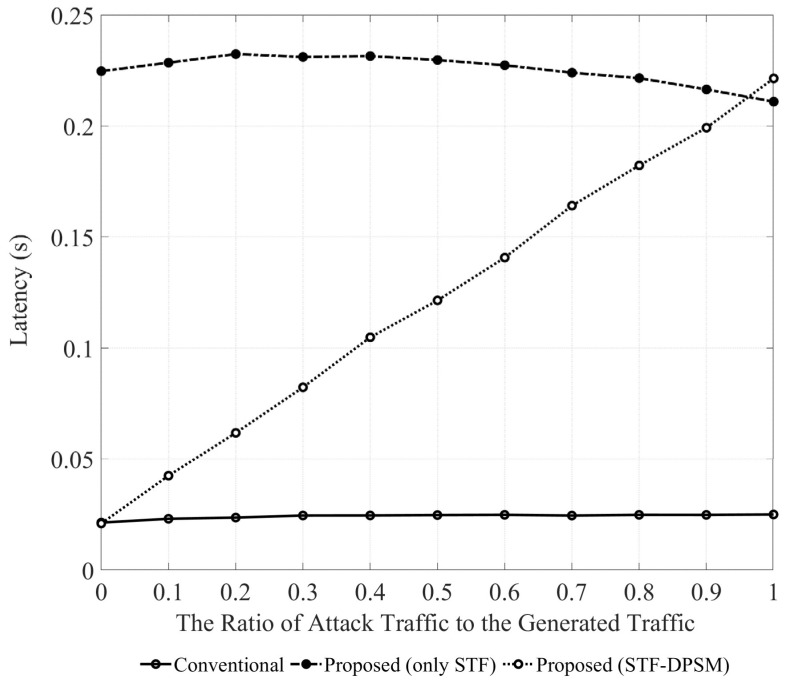
Latency based on the proportion of attack traffic in the total generated traffic.

Figure 8 illustrates the impacts of the conventional and proposed methods on latency based on the proportion of attack traffic within the total generated traffic. The conventional method maintains a constant traffic processing time, unaffected by the proportion of attack traffic, assuming an ideal channel environment without errors or interference. Furthermore, the method using only the STF experiences an increase in latency, due to the additional time required for consistent encryption/decryption and integrity checks regardless of traffic type, leading to higher overheads. The STF always incurs an additional overhead of 24 bytes and requires extra time for encryption/decryption and integrity checks, leading to a higher computational overhead compared to that of regular trigger frames without security. Therefore, the method that uses only the STF faces a trade-off between security and efficiency. The STF-DPSM method, which activates power saving mode upon detection of attack traffic and employs the STF only during the service period, also sees an increase in overheads as the proportion of attack traffic grows. However, in normal situations without attacks, the STF is not used, thereby reducing computational overheads and minimizing performance degradation. The method that consistently uses the STF increases latency by approximately 833% on average compared to the conventional approach. However, despite the increase, the STF-DPSM method, while exhibiting higher latency compared to the conventional method without defense mechanisms, achieves an average improvement of approximately 44.7% in latency compared to the method that consistently uses the STF, balancing the trade-offs between security and performance.

## 6. Conclusions

Wireless LAN-based IoT devices are often lightweight and powered by coin cell batteries, which presents challenges due to the high efficiency and performance demands of wireless LAN technology leading to excessive battery consumption. The trigger frame-based uplink transmission method, developed to improve network throughput, is deficient in security mechanisms, making it possible for attackers to tamper with trigger frames. Consequently, devices that receive these manipulated trigger frames respond immediately, which escalates power consumption. This issue becomes even more pronounced in next-generation devices that support multi-link operation, as they are more vulnerable to battery drain attacks compared to conventional single-link devices. This paper proposes an STF-DPSM specifically designed to counteract trigger frame-based battery drain attacks in multi-link environments. Experimental results demonstrate that the proposed method significantly enhances energy efficiency, achieving an average improvement of approximately 55.69% over conventional methods in scenarios with two links and a maximum power-saving time of 2^100^. Although the proposed method introduces an increase in latency compared to the conventional approach, it effectively reduces latency by approximately 44.7% on average compared to techniques that consistently perform encryption and integrity checks. This balance between enhanced security and maintained efficiency highlights the effectiveness of the proposed solution in mitigating energy consumption issues while ensuring robust security in multi-link wireless LAN environments.

In future research, we plan to evaluate the STF-DPSM method and other existing solutions under various network conditions in a realistic environment. Additionally, as the proposed method is a passive approach that detects battery drain attacks at normal APs, we intend to incorporate an active method to detect battery drain attacks directly on victim devices. We plan to evaluate a system that selectively applies either the passive or active approach in complex network environments.

## Figures and Tables

**Table 2:** System parameters.

Parameter	Meaning	Value
V	Voltage	1.1 V
T_TOTAL_	Total Transmission Time	-
T_TX_	Time spent in TX mode	-
T_RX_	Time spent in RX mode	-
T_IDLE_	Time spent in IDLE mode	-
T_DOZE_	Time spent in DOZE mode	-
T_SIFS_	SIFS	16 us
T_TWT_	TWT	variable
L_ACK_	Length of ACK	14 B
L_TF_	Length of Trigger Frame	34 B
L_STF_	Length of Secure Triggering Frame	58 B
L_PPDU_	Length of PPDU	1024 B
P_TX_	Power consumption in TX mode	308 mW
P_RX_	Power consumption in RX mode	110 mW
P_IDLE_	Power consumption in IDLE mode	55 mW
P_DOZE_	Power consumption in DOZE mode	0.0033 mW

## Data Availability

The data supporting the results presented in this paper are currently not publicly available but may be obtained from the authors upon reasonable request.
